# Improve water quality of small water bodies

**DOI:** 10.1016/j.xinn.2025.100938

**Published:** 2025-04-29

**Authors:** Sijia Li, Dehua Mao, Jonathan M. Chase, Kaishan Song, Zongming Wang, Hong Yang

**Affiliations:** 1 Key Laboratory of Wetland Ecology and Environment, Northeast Institute of Geography and Agroecology, Chinese Academy of Sciences, Changchun 130102, P.R. China; 2 Department of Geography and Environmental Science, University of Reading, RG6 6AB Reading, UK; 3 German Centre for Integrative Biodiversity Research (iDiv) Halle-Jena-Leipzig, 04103 Leipzig, Germany; 4 Department of Computer Science, Martin Luther University-Halle Wittenberg, 06099 Halle (Saale), Germany

## Main text

Nearly a decade after China launched its landmark *Water Pollution Prevention and Control Action Plan*—known as the “Water Ten Measures”—significant improvements have been achieved in the quality of large lakes and reservoirs (>0.1 km^2^). However, a major blind spot persists in water governance: the protection and restoration of small water bodies, typically ranging from 0.03 to 0.1 km^2^.

Despite their small size, these water bodies are ecologically indispensable. Globally, they represent nearly half of all inland water bodies. Often embedded in densely populated or agriculturally intensive landscapes, they are more directly exposed to anthropogenic pressures than larger aquatic ecosystems. Their small catchments and limited dilution capacity make them especially vulnerable to nutrient pollution, eutrophication, and greenhouse gas emissions such as CO_2_, CH_4_, and N_2_O.^1^ Consequently, they can serve as early indicators of environmental stress, offering valuable insight into land-water interactions at the local scale.

In China, recent national-scale investigations show that nearly half of small lakes (0.1–1 km^2^) have experienced reduced water clarity, with transparency declining by approximately 0.5 m. These figures are troubling, particularly as they do not even include the smallest lakes and ponds (<0.1 km^2^), which are even more susceptible to degradation but remain excluded from national environmental monitoring schemes.

The decline in these small systems is not just an ecological concern; it also poses a challenge to achieving Sustainable Development Goals (SDGs), especially SDG 6 on clean water and sanitation and SDG 13 on climate action. Small water bodies store carbon, support freshwater biodiversity, and offer services such as groundwater recharge, local climate regulation, and community-level water access. Ignoring them can jeopardize both environmental and social sustainability.

Policy progress has been made. In April 2022, China introduced the *Specifications for Conservation and Management of Small Wetlands*, a new national standard aimed at improving the management of these ecosystems.^2^ However, the standard focuses largely on spatial protection and administrative classification, with limited emphasis on water quality monitoring or restoration practices. This is a missed opportunity to integrate water quality objectives into small wetland conservation.

To address this gap, small water bodies must be formally recognized in national water policy frameworks. This includes setting water quality standards, funding targeted restoration projects, and incorporating these systems into regular environmental monitoring programs. Importantly, research is needed to develop context-specific strategies for managing these diverse ecosystems. Collaboration among scientists, local governments, and non-governmental organizations (NGOs) is essential in designing inclusive, effective, and scalable solutions.

Protecting small water bodies is not merely an environmental concern—it is an essential step toward achieving national and global sustainability goals. Their small size should not diminish their significance. On the contrary, these vulnerable yet vital ecosystems may hold the key to safeguarding China’s freshwater future.

## Funding and acknowledgments

This work was funded by the 10.13039/501100001809Natural Science Foundation of China (no. U2342008).

## Declaration of interests

The authors declare that they have no conflict of interest.
